# Care management services at safety-net clinics in the United States

**DOI:** 10.3389/frhs.2025.1646788

**Published:** 2025-09-19

**Authors:** Joseph H. Joo, Anna M. Morenz, Michael D. Dang, Jennifer R. Cardin, Joshua M. Liao

**Affiliations:** ^1^ Department of Internal Medicine, University of Texas Southwestern Medical Center, Dallas, TX, United States; ^2^ Program on Policy Evaluation & Learning, Dallas, TX, United States; ^3^ Department of Medicine, University of Arizona College of Medicine, Tucson, AZ, United States

**Keywords:** safety-net, federally qualified health centers, rural health centers, care management, social determinants of health

## Abstract

Federally qualified health centers and rural health centers are key parts of the United States ambulatory safety-net care system. Medicare has sought to encourage care coordination at these safety-net clinics by reimbursing clinicians directly for delivering care management services. In this paper, we described long-term trends in utilization of care management services for Medicare patients at federally qualified health centers and rural health centers vs. non-federally qualified health centers and non-rural health centers. General care management service utilization increased by 207% with 2,251 services submitted in 2023. Denial rates for general care management services increased over time, with 42% of submitted services being denied in 2018, compared to 64% of submitted services being denied in 2023. Compared to general care management services, transitional care management services were delivered far less frequently at federally qualified health centers and rural health centers, with a total of 237 services submitted across the entire six-year study period, and zero services submitted in several study years. Among these services, 188 (79%) were reimbursed with a corresponding cost of $31,551. Despite their greater salience and need for care coordination at safety-net clinics in the United States, there was little utilization of care management services delivered to Medicare patients and reimbursed through the physician fee schedule. The low uptake may reflect a preference for care management services administered outside of Medicare.

## Introduction

Federally qualified health centers (FQHCs) and rural health centers (RHCs) are key parts of the United States ambulatory safety-net care system. FQHCs and RHCs are located in medically underserved areas or health professional shortage areas that serve populations such as low-income, homeless, and rural Americans, including those insured through Medicare (1–5). In particular, FQHCs and RHCs are safety-net clinics that can coordinate care to manage transitions following acute care episodes and address drivers of chronic disease on an ongoing basis (6–8). Together, approximately 7,000 FQHCs and RHCs serve nearly 70 million patients annually, including over 4 million Medicare patients each year (3, 5, 9).

Indeed, care coordination for transitions of care and chronic disease management have been shown to improve patient outcomes. Prior studies have shown care coordination to reduce readmissions by about 5–6 percent and health care costs by approximately 8–14 percent, while also increasing the likelihood of treatment adherence by 10–16 percent (10–17). Similarly, studies have shown that behavioral health integration into primary care can reduce hospitalizations by about 37 percent, while increasing screening and treatment for depression by nearly 46 percent (18–20). In turn, over the last decade, as the largest payer nationwide, Medicare has sought to encourage care coordination by using the physician fee schedule – a comprehensive list of fees used by Medicare to directly reimburse clinicians on a fee-for-service basis – to deliver care management services, including ongoing coordination of chronic disease; coordination during transitions of care after acute hospitalization; and coordination between behavioral and physical health needs (21–26).

Federal policymakers would benefit from insight about if and how these services impact Medicare patients receiving care at safety-net clinics. However, despite this major national investment in care management services, and their potential benefit, little is known about how they have been used within safety-net clinic. Therefore, the primary objective of this study was to describe the long-term trends in utilization of care management services for Medicare patients at FQHCs and RHCs across the United States.

## Methods

Care management services of interest included principal care management (PCM; involving coordination of care for patients with a single chronic condition), chronic care management (CCM; involving coordination of care for patients with multiple chronic conditions), transitional care management (TCM; involving coordination of care for patients' post-hospitalization), and behavioral health integration (BHI; involving the coordination of mental or behavioral health care) (21–26). This analysis involved 2018–2023 Medicare claims including all professional services billed to and reimbursed by Medicare (27). We chose this timeframe to encompass a period in which care management services of interest were reimbursable by clinicians at FQHCs and RHCs.

We identified care management services using Current Procedural Terminology (CPT) codes, G codes (codes used by Medicare to reimburse clinicians for services lacking a CPT code), and place of service modifiers. Given evolution over time in coding guidance provided to clinicians (e.g., which CPT or G codes to use for which care management services), we analyzed trends in utilization over time by combining PCM, CCM, and BHI services into a category of *general care management* (GCM) services. Combining these reflects how G code (G0511) grew to encompass PCM, CCM, and BHI services at FQHCs and RHCs since 2021. Because TCM services were not included in the GCM services (G0511) used throughout FQHCs and RHCs, TCM services were analyzed separately using codes 99495 and 99496. The methods to identify care management services were consistent with prior studies using CPT codes and place of service modifiers to describe costs and utilization (28–32).

We compared care management services at FQHCs/RHCs (place of service modifiers 50 and 72) vs. non-FQHCs/RHCs (i.e., general clinics; all other place of service modifiers). For both FQHCs/RHCs and non-FQHCs/RHCs, we calculated the cumulative sum of submitted, reimbursed, and denied services, along with associated costs. Our analysis was completed using Python version 3.12.3 (Packages: Pandas 2.2.2) and followed Consolidated Health Economic Evaluation Reporting Standards reporting guidelines where applicable. Given the publicly available nature of all study data, the University of Texas Southwestern Medical Center institutional review board waived approval per institutional policy.

## Results

Across FQHCs and RHCs, general care management service utilization increased by 207%, with 733 services submitted in 2018 compared to 2,251 services submitted in 2023 (Table 1). Denial rates for general care management services increased over time, with 42% of submitted services being denied in 2018, compared to 64% of submitted services being denied in 2023. Reimbursed general care management services represented $15,861 in spending in 2018 and $50,912 in 2023.

**Table 1:** Utilization of care management services at federally qualified health centers and rural health centers compared to non-federally qualified health centers and non-rural health centers from 2018-2023. Utilization includes services submitted, services allowed, submitted services cost, and allowed services cost.

		FQHCs/RHCs				Non-FQHCs/RHCs			
	Year	Submitted services, no.	Submitted payments, $	Allowed services, no.	Allowed payments, $	Submitted services, no.	Submitted payments, $	Allowed services, no.	Allowed payments, $
GCM^a^	2018	733	$58,233	427	$15,861	4,976,638	$372,867,668	4,693,358	$209,278,362
	2019	533	$40,237	433	$15,304	5,396,463	$423,680,624	5,113,605	$233,003,831
	2020	1,506	$168,202	306	$11,811	5,955,597	$493,915,822	5,673,299	$259,321,618
	2021	2,057	$244,412	503	$18,299	6,739,659	$581,686,228	6,361,798	$280,797,337
	2022	1,525	$169,662	410	$20,708	8,053,187	$819,354,391	7,593,521	$476,965,612
	2023	2,251	$229,843	807	$50,912	11,534,507	$1,308,622,682	10,668,509	$657,587,361
TCM^b^	2018	0	$0	0	$0	1,328,769	$477,234,290	1,272,524	$238,364,031
	2019	0	$0	0	$0	1,488,140	$545,608,223	1,422,828	$265,381,454
	2020	116	$35,320	116	$18,603	1,171,689	$446,943,801	1,143,743	$230,896,764
	2021	86	$24,426	72	$12,948	1,230,538	$496,961,847	1,199,267	$271,265,771
	2022	0	$0	0	$0	1,240,785	$523,627,504	1,198,776	$271,301,610
	2023	35	$4,489	0	$0	1,343,520	$584,437,071	1,303,122	$289,657,681

^a^ GCM, General Care Management = PCM, CCM, and BHI. PCM Codes: G0511 + 99,424, 99,425, 99,426, 99,427, G2064, and G2065. CCM Codes: G0511 + 99,487, 99,489, 99,490, 99,491, 99,439, 99,437, G0506, G3002, G3002, GCCC1. BHI Codes: G0511 + 99,484, 99,492, 99,494, G0323, G2214, G0502, G0503, and G0507.

^b^ TCM, Transitional Care Management. TCM Codes: 99,495 and 99,496.

Compared to general care management services, TCM services were delivered far less frequently at FQHCs and RHCs, with a total of 237 services submitted across the entire six-year study period, and zero services submitted in several study years. Among these services, 188 (79%) were reimbursed with a corresponding cost of $31,551.

Outside of FQHCs and RHCs, general care management service utilization increased over time. In particular, a total of 4,976,638 such services were submitted in 2018, compared to 11,534,507 total services were submitted in 2023 (146% increase). The majority of submitted general care management services (94%) were reimbursed across the study period, corresponding to a denial rate of 6%. The 4,693,358 reimbursed services accounted for $209,278,362 in 2018 and 10,668,509 reimbursed services accounted for $657,587,361 in 2023.

In non-FQHCs and non-RHCs, TCM utilization remained consistent over time, trending from 1,328,769 to 1,343,520 services submitted from 2018 to 2023, respectively. Across the study period, the majority of TCM services (97%) were reimbursed: 1,272,524 services (corresponding to $238,364,031) in 2018 and 1,303,122 services (corresponding to $289,657,681) in 2023.

## Discussion

Despite the need to coordinate care for patients receiving care at safety-net clinics in the US, care management services available through national physician fee schedule were infrequently delivered to Medicare patients in these clinics. While there are fewer safety-net clinics across the US compared to other clinics – approximately 7,000 FQHCs/RHCs compared to over 50,000 clinics (1, 2, 33) – the difference in utilization is disproportional to the ratio of safety-net and general clinics. Furthermore, despite increases in federal funding to FQHCs/RHCs during the pandemic period and temporary waivers expanding telehealth coverage, care management services delivered at safety-net clinics increased modestly between 2020 and 2023 (34, 35).

Low utilization could arise from several factors. For one, Medicare patients receiving care at safety-net clinics may be dually eligible for Medicaid, and clinic staff may coordinate care via services reimbursed through Medicaid (e.g., providing coordination activities included in Medicaid managed care contracts) rather than services reimbursed through Medicare (36–39). For another, safety-net clinics may use usual clinic visits, rather than care management services to coordinate care. While generalizable data on these dynamics are sparse, anecdotal experience suggests they could contribute at least partially for explaining low use of care management services observed in this analysis.

The implication of such dynamics would be several fold. First, safety-net clinic preference for using Medicaid rather than Medicare care management services would potentially highlight several deficiencies in the latter. For instance, safety-net clinics in some states can provide care management services reimbursed through Medicaid managed care organizations. Some of these Medicaid-reimbursed services focus on and encompass both clinical and non-clinical drivers of health (i.e., social determinants of health), a scope that extends beyond the scope of PCM and CCM, which focus on clinical drivers. Such care management services can be reimbursed on a regular, prospective “per member per month” basis, compared to the retrospective per service basis on which PCM and CCM are reimbursed. Additionally, the financial incentive for safety-net clinics to utilize care management rather than the evaluation and management services may not be practical considering the number of administrative requirements.

In contrast, Medicare care management services require clinicians to meet a strict set of criteria, posing potentially onerous implementation challenges or administratively burdensome requirements for providing and billing for these services (40). To that end, anecdotally some clinics have contracted third parties to alleviate the administrative burden (41–43) – a find that comports with our finding that 65% of Medicare care management services provided and billed by safety-net clinics were denied and not reimbursed. Ultimately, it may be difficult to encourage adoption of Medicare care management services if they encourage incomplete focus on drivers of health facing safety-net populations (e.g., clinical but not non-clinical determinants of health), increase administrative burden, and provide retrospective reimbursement; and if less onerous and more easily implementable alternatives (e.g., Medicaid care management services) exist.

A second, and related, implication of the potential dynamics described above is that national health care and policy leaders have limited insight into the strategies through which care is coordinated for underserved Medicare patients in the outpatient setting. Part of the appeal of separately billed, stand-alone care management services such as PCM, CCM, BHI, and TCM is that they provide a way for leaders to understand how and what types of care coordination can improve outcomes. If safety-net clinics opt to use non-Medicare payer services or regular services instead to coordinate care, Medicare will have incomplete information about care coordination and its impact of outcomes.

Policymakers can take several steps to address these issues. First, they can take steps to improve data collection to improve the visibility of the nature and extent of care management services provided to Medicare patients, regardless of whether such services are provided through Medicare services (e.g., TCM, CCM) or other avenues (e.g., Medicaid managed contract arrangements). This goal could be achieved by requiring that clinics such as FQHCs report this information alongside documentation they already provide for reimbursement through the Prospective Payment System. Second, policymakers can reform care coordination services to reduce sources of administrative burden or confusion, for instance those that arise from documentation requirements around patient consent, patient care plan in certified electronic health records, and time thresholds. Such changes are feasible in partially underway with the creation and promotion of Advanced Primary Care Management services that build on TCM and CCM while shifting from billing for time-based activity to billing patient per month. Third, policymakers could create new avenues for safety-net clinicians to address social determinants of health to address social determinants of health through the fee schedule. For example, in 2024, policymakers incorporated community health integration into the general care management at FQHCs and RHCs reflecting a shift to value and integrate addressing social determinants of health in patient care (44). Future reforms could extend such efforts, integrating screening for social determinants of health into existing care management and community health integration services.

Study limitations included descriptive nature and data limitations of aggregate-level CPT/G codes, which precluded patient- and geographic-level results. Future work must build on our analysis, which provides timely insight about the dearth of care management services among safety-net clinics and identify potential strategies for overcoming implementation and billing barriers. This work is urgently needed, especially with continued growth in the need for care coordination among groups such as underserved Medicare patients and the emergence of new care coordination codes for meeting that need.

## Data Availability

The original contributions presented in the study are included in the article/Supplementary Material, further inquiries can be directed to the corresponding author.
